# Streptococcus pyogenes Hijacks Host Glutathione for Growth and Innate Immune Evasion

**DOI:** 10.1128/mbio.00676-22

**Published:** 2022-04-25

**Authors:** Stephan Brouwer, Magnus G. Jespersen, Cheryl-lynn Y. Ong, David M. P. De Oliveira, Bernhard Keller, Amanda J. Cork, Karrera Y. Djoko, Mark R. Davies, Mark J. Walker

**Affiliations:** a Australian Infectious Diseases Research Centre and School of Chemistry and Molecular Biosciences, The University of Queensland, St. Lucia, Australia; b Department of Microbiology and Immunology at the Peter Doherty Institute for Infection and Immunity, The University of Melbourne, Melbourne, Victoria, Australia; c Department of Biosciences, Durham University, Durham, United Kingdom; Nanyang Technological University

**Keywords:** *Streptococcus pyogenes*, oxidative stress, redox homeostasis, virulence regulation, immune evasion, glutathione uptake

## Abstract

The nasopharynx and the skin are the major oxygen-rich anatomical sites for colonization by the human pathogen Streptococcus pyogenes (group A Streptococcus [GAS]). To establish infection, GAS must survive oxidative stress generated during aerobic metabolism and the release of reactive oxygen species (ROS) by host innate immune cells. Glutathione is the major host antioxidant molecule, while GAS is glutathione auxotrophic. Here, we report the molecular characterization of the ABC transporter substrate binding protein GshT in the GAS glutathione salvage pathway. We demonstrate that glutathione uptake is critical for aerobic growth of GAS and that impaired import of glutathione induces oxidative stress that triggers enhanced production of the reducing equivalent NADPH. Our results highlight the interrelationship between glutathione assimilation, carbohydrate metabolism, virulence factor production, and innate immune evasion. Together, these findings suggest an adaptive strategy employed by extracellular bacterial pathogens to exploit host glutathione stores for their own benefit.

## INTRODUCTION

Glutathione is a tripeptide consisting of the three amino acids glutamate, cysteine, and glycine. It is the most abundant low-molecular-weight thiol compound present at millimolar concentrations in almost all eukaryotic cells, where it plays key roles in antioxidant defense, nutrient metabolism, and redox signaling (1). The cysteine thiol group in glutathione is responsible for this biological activity, the primary function of which is to maintain the intracellular redox homeostasis, protecting cells against oxidative damage by donating reducing equivalents directly to electrophilic compounds, such as oxygen radical species, or to glutathione peroxidase. Glutathione peroxidase is a cytosolic enzyme which catalyzes detoxification of hydrogen peroxide (H_2_O_2_) and lipid peroxides, resulting in the formation of oxidized glutathione (GSSG). The flavoenzyme glutathione reductase (GR) restores intracellular levels of the reduced and active form of glutathione (GSH) by reducing GSSG using NADPH as an electron donor, thereby maintaining the cellular supply of GSH. As a result, the GSH/GSSG ratio can be considered a cellular redox sensor that determines the oxidative status of the cell. Consequently, the GSH/GSSG ratio is homeostatically maintained by the activity of these two enzymes (2).

Despite this key role in protecting eukaryotic cells against oxidative toxicity, glutathione biosynthesis in bacteria is largely confined to Gram-negative proteobacteria and cyanobacteria through the action of the two canonical enzymes, γ-glutamylcysteine synthetase (GshA) and glutathione synthetase (GshB) (3, 4). Remarkably, only a few Gram-positive bacteria are capable of producing glutathione. In these Gram-positive bacteria, the classical two-step biosynthesis of glutathione is absent and is carried out by a single bifunctional fusion protein GshF (5, 6). Most Gram-positive bacteria that lack *de novo* glutathione biosynthesis produce functionally analogous cysteine derivatives. These include the sugar-based cysteinyl derivatives mycothiol (MSH), which is present in *Actinobacteria* (7), and bacillithiol (BSH), which is produced by *Bacillus* species and some members of the genera Staphylococcus and Streptococcus (8). In addition, glutathione import has emerged as an alternative strategy employed by some *Firmicutes* to accumulate intracellular glutathione pools (8–11). For instance, the glutathione binding protein GshT has been identified as part of a cellular glutathione transport machinery in Streptococcus mutans and Streptococcus pneumoniae (10, 11).

The human-adapted pathogen Streptococcus pyogenes (group A Streptococcus [GAS]) is one of a few streptococcal species that accumulates significant levels of glutathione (9) despite the absence of a *de novo* glutathione biosynthetic pathway. GAS encodes several glutathione-dependent enzymes, including glutathione peroxidase (GpoA), which contributes to GAS virulence in a systemic infection model (12). Intracellular GSH levels are determined by the ability of GAS to salvage glutathione from the environment (13). The relative importance of the glutathione salvage pathway during GAS infection is largely unexplored, but recent evidence suggests that intracellular glutathione is critical for maintaining metal ion homeostasis (14). While highly abundant in the host cytosol, glutathione is present at much lower concentrations in the more oxidizing extracellular environment (15). We have recently reported that host cell damage by the cholesterol-dependent cytolysin streptolysin O (SLO) allows GAS to gain access to host cytosolic glutathione stores (16). Here, the ABC transporter substrate binding protein GshT is identified as integral for GAS glutathione import. We report a key role for host-derived glutathione in GAS aerobic metabolism and show that glutathione accumulation is linked to the expression of virulence factors. Further, glutathione is demonstrated as an essential metabolite required for resistance to oxidative stress, enabling GAS to evade host innate immunity.

## RESULTS

### GshT facilitates glutathione import in GAS and is critical for aerobic glycolysis.

**FIG 1:**
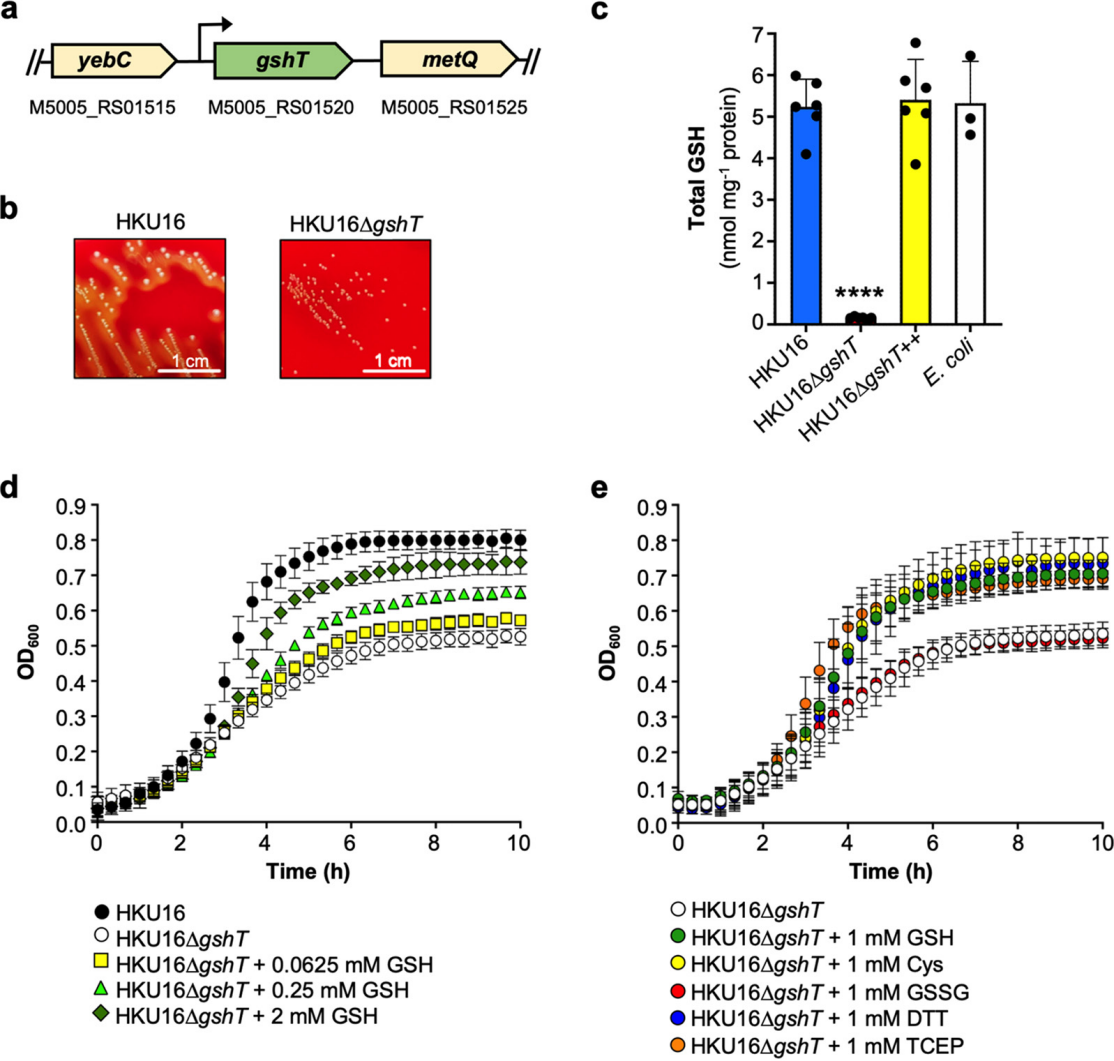
GshT is required for glutathione import and aerobic growth. (a) Schematic of the *gshT* open reading frame in GAS. The accession number of the reference serotype M1 strain MGAS5005 (GenBank accession no. NC_007297.2) is given for each gene. The predicted *gshT* promoter is indicated (arrow) (17). (b) Colony morphology of HKU16 and HKU16Δ*gshT* on 5% horse blood agar after overnight incubation at 37°C. (c) Intracellular accumulation of glutathione in cell lysates of indicated strains (*n* = 6) from overnight growth on horse blood plates. Total GSH is presented as the sum of GSSG plus GSH. Statistical significance was assessed using one-way ANOVA with Dunnett’s multiple comparisons *post hoc* test against the HKU16 wild-type control group (****, *P < *0.0001 for HKU16Δ*gshT*). Similar results were obtained from E. coli lysates under the same growth conditions (*n* = 3), which served as a control group for glutathione measurements. (d) Growth curves of indicated strains in THY medium supplemented with 0.0625, 0.25 and 2 mM GSH (*n* = 3). (e) Growth curves of HKU16Δ*gshT* in THY medium supplemented with 1 mM indicated reducing agents (*n* = 3). All data are presented as mean values ± SD.

The putative *gshT* homologous gene product of GAS shares 61% and 51% amino acid sequence identity with GshT from S. mutans (SMU.1942c; GenPept accession no. AAN59552.1) (17) and S. pneumoniae (SPD_0150; GenPept accession no. AVN85326.1) (11), respectively (Fig. S1 in the supplemental material). In the GAS genome, the *gshT* homologue is a standalone gene flanked by a putative transcriptional regulatory gene (*yebC*) and a putative *metQ* family ABC transporter substrate binding protein (Fig. 1a). To determine the potential role of *gshT* in glutathione acquisition, we generated an isogenic knockout mutant strain in the GAS *emm*12 scarlet fever isolate HKU16 (16, 18). Mutation of *gshT* had a profound impact on the colony morphology of HKU16, resulting in a much smaller and hypohemolytic colony phenotype on blood agar plates (Fig. 1b), without affecting bacterial cell size and shape (Fig. S2). To confirm that the *gshT* gene encodes a functional component of a glutathione importer, intracellular glutathione levels (total GSH = oxidized glutathione [GSSG] + reduced glutathione [GSH]) were quantified in bacterial lysates from overnight growth on blood plates. The HKU16 wild-type strain accumulated approximately 5 nmol total GSH per mg protein, whereas glutathione was undetectable in the HKU16Δ*gshT* knockout mutant (Fig. 1c). Genetic complementation of HKU16Δ*gshT* with the wild-type *gshT* gene (HKU16Δ*gshT*^++^) fully restored wild-type glutathione abundance. Interestingly, total GSH levels in cell lysates of Escherichia coli were similar to levels detected in HKU16 lysates (Fig. 1c), demonstrating that glutathione salvage in GAS generates an intracellular total GSH pool similar in magnitude to that produced by *de novo* glutathione biosynthesis in E. coli under these conditions.

10.1128/mbio.00676-22.1 FIG S1 Multiple-sequence alignment (Clustal Omega) of GshT from S. pyogenes (Spy), S. mutans (Smu), and S. pneumoniae (Spn). Download FIG S1, TIF file, 2.1 MB. Copyright © 2022 Brouwer et al. 2022 Brouwer et al. https://creativecommons.org/licenses/by/4.0/ This content is distributed under the terms of the Creative Commons Attribution 4.0 International license.

10.1128/mbio.00676-22.2 FIG S2 Scanning electron microscopy images of HKU16 wild-type and HKU16Δ*gshT* grown overnight in THY at 37°C. Download FIG S2, TIF file, 0.3 MB. Copyright © 2022 Brouwer et al. 2022 Brouwer et al. https://creativecommons.org/licenses/by/4.0/ This content is distributed under the terms of the Creative Commons Attribution 4.0 International license.

To better understand the physiological role of glutathione utilization in GAS, we next analyzed *in vitro* growth kinetics of HKU16Δ*gshT* in nutrient-rich medium (Todd-Hewitt yeast extract [THY]) under aerobic conditions. Under these conditions, growth rates of both HKU16Δ*gshT* and wild type were similar during the early logarithmic growth phase, but the mutant showed a marked growth defect at later stages of growth and was unable to reach a culture density equivalent to that of the wild type (Fig. 1d). GSH supplementation was sufficient to restore growth of HKU16Δ*gshT* in a dose-dependent manner, with a growth rate comparable to that of the wild-type strain at a concentration of 2 mM GSH (Fig. 1d). Since GSH is impermeable to HKU16Δ*gshT* (Fig. 1c), it is possible that GSH supplementation quenches extracellular reactive oxygen species (ROS) produced by GAS during aerobic growth (19), representing a potential self-limiting factor for HKU16Δ*gshT* growth (Fig. 1b and d). To test this hypothesis, we initially grew HKU16Δ*gshT* in the presence of l-cysteine. Like GSH, the addition of l-cysteine restored growth of the mutant strain. Supplementation with the two reducing agents dithiothreitol (DTT) and Tris(2-carboxyethyl)phosphine-HCl (TCEP), a non-thiol-reducing agent which is generally impermeable to cell membranes (20), similarly restored growth of HKU16Δ*gshT*. In contrast, GSSG supplementation had no effect on the growth pattern of HKU16Δ*gshT* (Fig. 1e). These findings were validated using a chemically defined medium (CDM) with glucose as the sole carbon source for growth (16). Such conditions are more likely to resemble the nutrient-poor extracellular environment of GAS infection, where the *gshT* mutant exhibited growth kinetics identical to those of wild type in the presence of 0.2 mM reducing agents (Fig. S3). In contrast to growth in THY medium, HKU16Δ*gshT* was unable to grow in CDM in the absence of reducing agent supplementation. Collectively, these data demonstrate that GshT-mediated import of glutathione is essential for aerobic growth of GAS and that a shift in the extracellular redox potential is sufficient to compensate for the loss of *gshT*.

10.1128/mbio.00676-22.3 FIG S3 Growth curves of indicated HKU16 strains in THY medium supplemented with 12.5, 25, 50, 100, and 200 μM of indicated reducing agents (*n* = 1). Download FIG S3, TIF file, 1.1 MB. Copyright © 2022 Brouwer et al. 2022 Brouwer et al. https://creativecommons.org/licenses/by/4.0/ This content is distributed under the terms of the Creative Commons Attribution 4.0 International license.

### Metabolic redox dynamics in GAS.

In response to oxidative stress, eukaryotic cells redirect their metabolic flux from glycolysis to the oxidative pentose phosphate pathway (oxPPP) (21, 22), an alternative pathway to glycolysis. The first and rate-limiting enzyme of the oxPPP is glucose-6-phosphate dehydrogenase (G6PD) which generates NADPH, the major source of reducing equivalents for the protection of cells against oxidative injury. In bacteria, the oxPPP and the tricarboxylic acid (TCA) cycle are considered the major sources of NADPH (23). However, as a result of reductive evolution, GAS does not encode a functional TCA cycle and is also unable to execute the oxPPP due to the absence of glucose-6-phosphate dehydrogenase, 6-phosphogluconolactonase, and phosphogluconate dehydrogenase (24).

**FIG 2:**
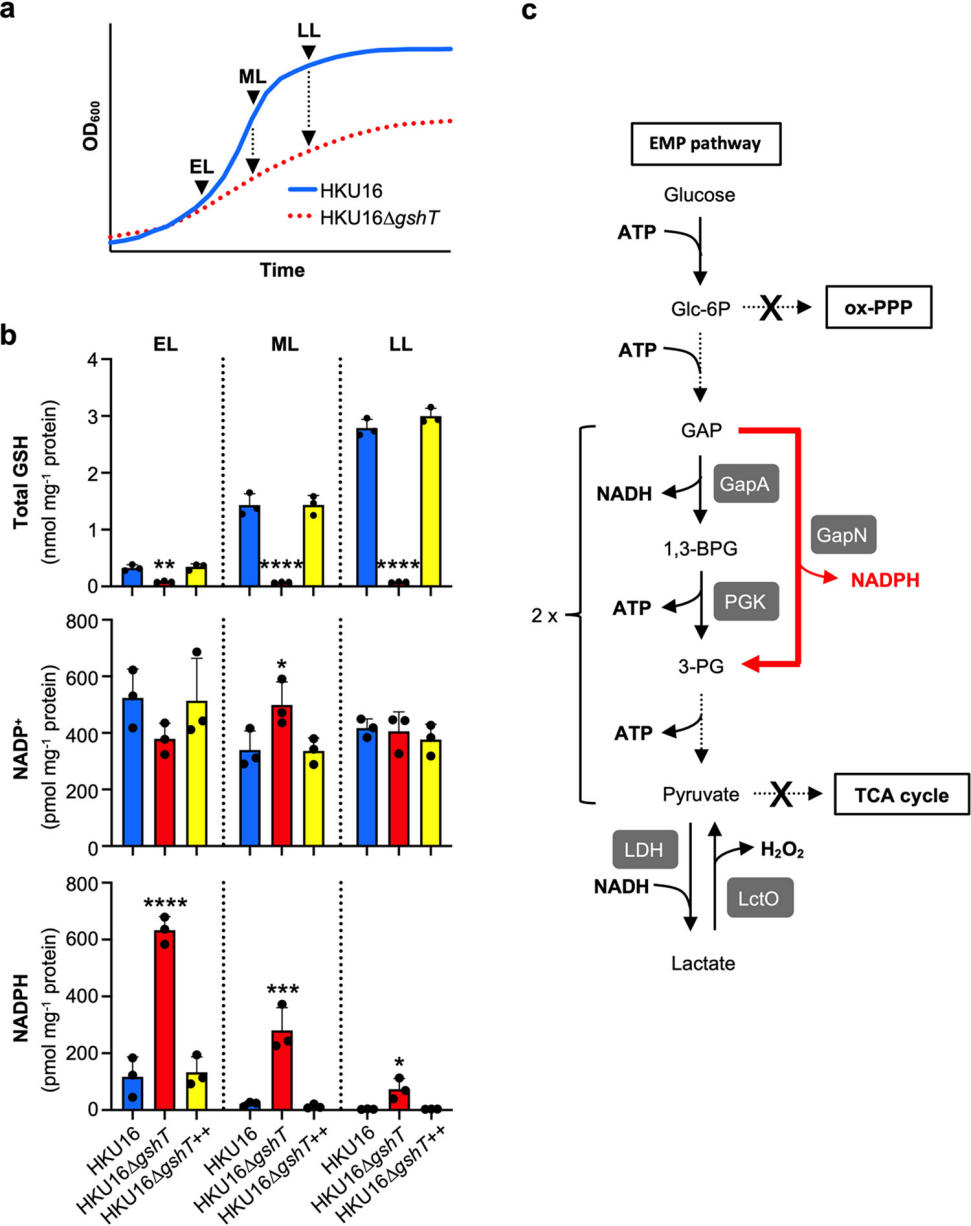
Role of glutathione in metabolic redox changes. (a) Schematic representation of the sampling time points at early logarithmic (EL), mid-logarithmic (ML), and late logarithmic (LL) growth phases to measure intracellular redox couple abundances. (b) Quantification of intracellular levels of total GSH and NADP(H) in indicated strains (*n* = 3). Statistical significance was assessed using one-way ANOVA with Dunnett’s multiple comparisons *post hoc* test against the HKU16 wild-type control group (*, *P < *0.0; **, *P < *0.01; ***, *P < *0.001; ****, *P < *0.0001). All data are presented as mean values ± SD. Source data are provided as a source data file. (c) The Embden-Meyerhof-Parnas (EMP) glycolytic pathway in GAS. Branching points for the oxidative pentose phosphate pathway (ox-PPP) and the tricarboxylic acid (TCA) cycle are shown. Enzymes of interest, namely, GapA (NAD^+^-dependent GAPDH [glyceraldehyde-3-phosphate dehydrogenase]; M5005_RS01330), PGK (phosphoglycerate kinase; M5005_RS07900), GapN (NADP^+^-dependent GAPDH; M5005_RS05535), LDH (lactate dehydrogenase; M5005_RS04370), and LctO (lactate oxidase; M5005_RS01875) are shown.

To study possible adaptations of GAS to cope with glutathione depletion, we analyzed redox-related metabolic dynamics in the *gshT* mutant strain grown in THY medium. Total GSH levels and concentrations of the NADP^+^/NADPH redox couple were quantified in whole-cell lysates, which were prepared at different time points of growth (early logarithmic, mid-logarithmic, and late logarithmic growth phase) (Fig. 2a). As expected, HKU16Δ*gshT* was unable to import glutathione from the growth medium, whereas total GSH gradually accumulated with up to 3 nmol per mg protein at late logarithmic growth phase in the wild-type strain, with significant differences already detectable at early logarithmic growth phase (Fig. 2b). Intracellular NADPH levels were much more abundant in HKU16Δ*gshT*, with the highest concentrations detectable at the early logarithmic growth phase (~600 pmol per mg protein), remarkably resembling a eukaryotic cellular response to oxidative stress. In contrast to total GSH levels in the wild type, the NADPH pool gradually decreased over time in the *gshT* mutant strain. Comparable amounts of the oxidized form (NADP^+^) were detected across the different growth phases, remaining at a constant level of ~400 to 500 pmol per mg protein. There were no major differences observed for the NAD^+^/NADH redox couple, albeit slightly elevated NADH levels were detected in HKU16Δ*gshT* at early logarithmic growth phase (Fig. S4a). Importantly, the complemented mutant strain HKU16Δ*gshT*^++^ exhibited a metabolic profile similar to that of the wild-type strain. We also quantified changes in the NADP^+^/NADPH and NAD^+^/NADH redox couples from growth obtained from blood plates. Intracellular NADPH levels were again significantly increased in HKU16Δ*gshT*, while no significant differences were detected for the NAD^+^/NADH redox couple (Fig. S4b and c), confirming previous results in THY. GAS encodes a functional nonphosphorylating glyceraldehyde-3-phosphate dehydrogenase (GapN; EC 1.2.1.9) (23–26), which irreversibly oxidizes glyceraldehyde-3-phosphate (GAP) to 3-phosphoglycerate (3-PG) (Fig. 2c). This alternative route of the classical Embden-Meyerhof-Parnas (EMP) pathway is an important NADPH-generating reaction in both GAS and S. mutans (26–28). We hypothesize that GapN may contribute to increased NADPH production in HKU16Δ*gshT*.

10.1128/mbio.00676-22.4 FIG S4 (a) Quantification of intracellular concentrations of the NAD(H) redox couple in indicated HKU16 strains (*n* = 3) grown in THY to early logarithmic (EL), mid-logarithmic (ML), and late logarithmic (LL) growth phase. (b and c) Intracellular levels of NADP(H) (b) and NAD(H) redox couples (c) in cell lysates of indicated HKU16 strains (*n* = 3) from overnight growth on horse blood plates. Statistical significance was assessed using one-way ANOVA with Dunnett’s multiple-comparison *post hoc* test against the HKU16 wild-type control group (*, *P < *0.05; ***, *P < *0.001; ****, *P < *0.0001 for HKU16Δ*gshT*; ns, not significant). Download FIG S4, TIF file, 0.5 MB. Copyright © 2022 Brouwer et al. 2022 Brouwer et al. https://creativecommons.org/licenses/by/4.0/ This content is distributed under the terms of the Creative Commons Attribution 4.0 International license.

### Glutathione coordinates the expression of metabolic and virulence genes in GAS.

**FIG 3:**
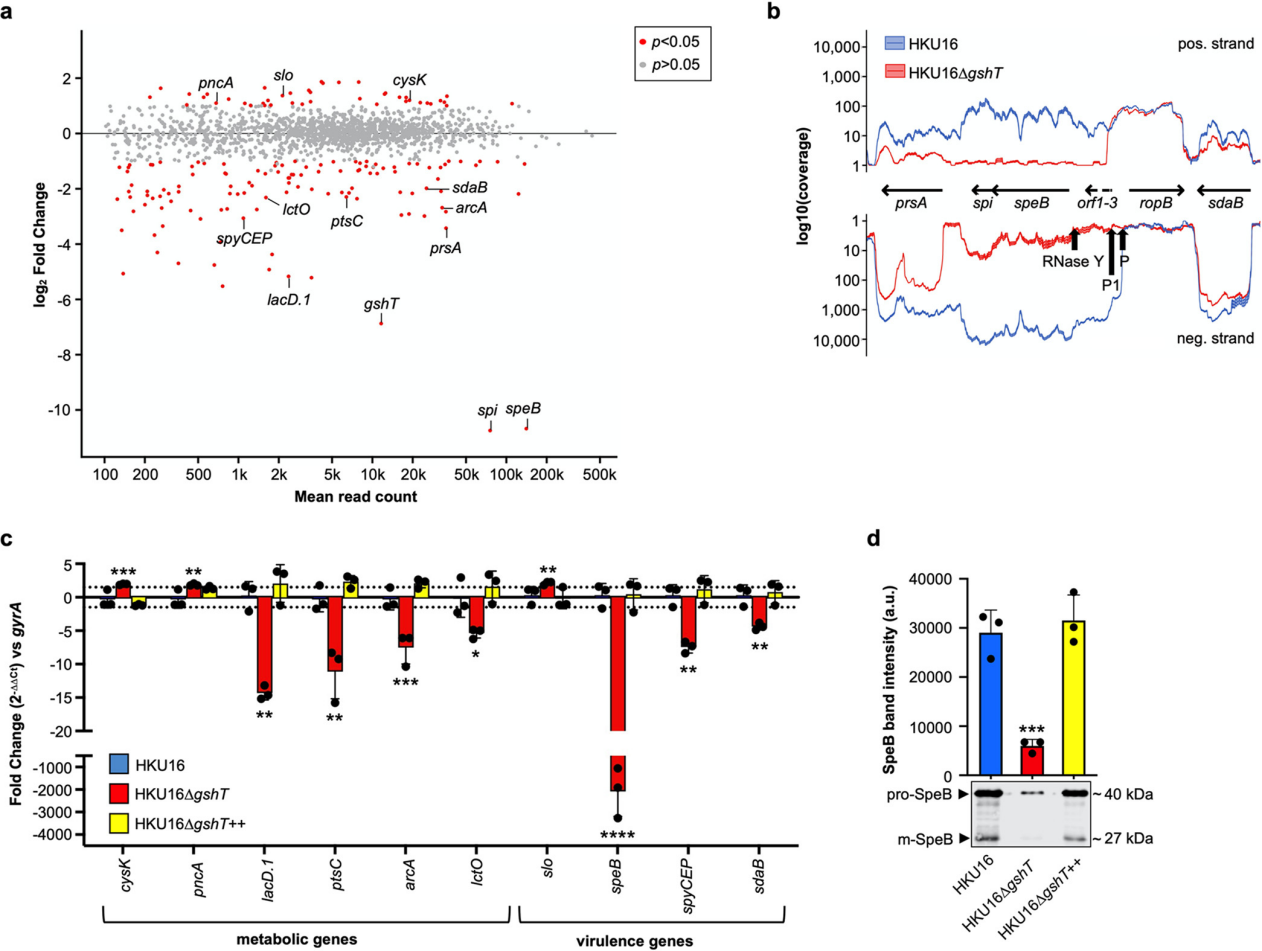
Global transcriptional response of GAS to glutathione depletion. (a) MA plot of the log_2_ fold change of all genes in HKU16Δ*gshT* compared to the HKU16 wild-type strain (*n* = 3). Green points indicate genes with a log_2_ fold change greater than 1.0 or less than −1.0 and *P* value of <0.05. (b) RNA-seq expression profile of the *speB*-*prsA* operon region in indicated strains. The plots illustrate the overall coverage distribution displaying the total number of sequenced reads. The two *speB* promoters P and P1, as well as the endoribonuclease Y (RNase Y) processing site, are indicated by black arrows (86). (c) Quantitative real-time PCR of select metabolic and virulence genes in indicated strains (*n* = 3). Data are presented as mean values ± SD. (d) Immunoblot detection of SpeB in culture supernatants of indicated strains. Band intensities of the zymogen (pro-SpeB) and mature (m-SpeB) form of SpeB were quantified with ImageJ. Data are presented as mean values ± SD. Statistical significance was assessed using one-way ANOVA with Dunnett’s multiple-comparison *post hoc* test against the HKU16 wild-type control group (***, *P* < 0.001 for HKU16Δ*gshT*) (*n* = 3).

Host glutathione can act as a spatiotemporal cue for some intracellular pathogens to switch on virulence gene circuits at the right time and environmental niche (29–34), prompting us to investigate whether GAS can also utilize host acquired glutathione as a signal to regulate virulence circuits. We examined global gene expression patterns in wild-type HKU16 and HKU16Δ*gshT* grown in THY to late logarithmic growth phase using transcriptome sequencing (RNA-seq). A total of 165 differentially regulated genes were identified in HKU16Δ*gshT* (Table S1) (Fig. 3a). Of these, 42 genes (25%) were upregulated, and 123 genes (75%) were downregulated in HKU16Δ*gshT*. Upregulated genes in HKU16Δ*gshT* included the gene encoding streptolysin O (*slo*) and the cysteine synthase A gene (*cysK*). SLO plays a role in the release of host cellular glutathione stores (26), while upregulation of cysteine synthesis may help maintain intracellular redox homeostasis (35). Additionally upregulated in the *gshT* mutant strain was the nicotinamidase gene *pncA*. PncA is a functionally confirmed nicotinamidase in GAS (36) and plays an important role in the NAD^+^ salvage and recycling pathway (37). Since GAS cannot *de novo* synthesize NAD^+^, it is dependent on salvage of the exogenous pyridine precursors, nicotinamide or nicotinic acid (vitamin B_3_) (36). This pathway ensures the supply of NAD^+^ into GAS, which can then be further reduced to NAD(P)H, providing a possible molecular explanation for increased NADPH production in HKU16Δ*gshT* under these growth conditions.

10.1128/mbio.00676-22.5 TABLE S1 Differentially regulated genes in HKU16Δ*gshT* at late logarithmic growth phase in THY grown at 37°C. Download Table S1, DOCX file, 0.03 MB. Copyright © 2022 Brouwer et al. 2022 Brouwer et al. https://creativecommons.org/licenses/by/4.0/ This content is distributed under the terms of the Creative Commons Attribution 4.0 International license.

Transcripts reduced in abundance in the HKU16Δ*gshT* mutant included virulence-determinant genes such as *speB*, encoding the cysteine protease streptococcal pyrogenic exotoxin B and adjacent cotranscribed genes (*spi*, a SpeB inhibitor; *prsA*, a peptidyl-prolyl isomerase involved in SpeB processing; and a hypothetical open reading frame [ORF] termed *orf*-3) (38) (Fig. 3b). Other downregulated virulence genes included *sdaB*, encoding streptodornase B SdaB/MF-1 (39), and *scpC*, encoding the interleukin 8 (IL-8) protease SpyCEP/ScpC (40). In addition, we detected reduced expression of several metabolic gene clusters, including the streptococcal *lac*.1 and *lac.*2 operons (41), the arginine deiminase operon (*arcABCD*) (42), as well as the mannose/fructose phosphoenolpyruvate-dependent phosphotransferase system (PTS) (43) in GAS. Transcript levels of the lactate oxidase gene (*lctO*) were also less abundant in the HKU16Δ*gshT* mutant. LctO catalyzes the oxidation of excess lactate generated from glucose by the EMP pathway to pyruvate and H_2_O_2_ (Fig. 2c) and is responsible for the generation of millimolar amounts of H_2_O_2_ under aerobic conditions (19, 44). Results of the RNA-seq-based transcriptome analysis were validated by quantitative real-time PCR of select genes (Fig. 3c). Furthermore, Western blot analysis of culture supernatants demonstrated that SpeB protein levels were significantly reduced in the HKU16Δ*gshT* mutant strain (Fig. 3d), correlating with reduced *speB* transcript abundance. Altogether, our data show that GAS glutathione acquisition plays a key role in shaping metabolic processes and that knockout of *gshT* alters the virulence factor expression profile of GAS.

### Glutathione protects GAS against ROS and neutrophil killing.

**FIG 4:**
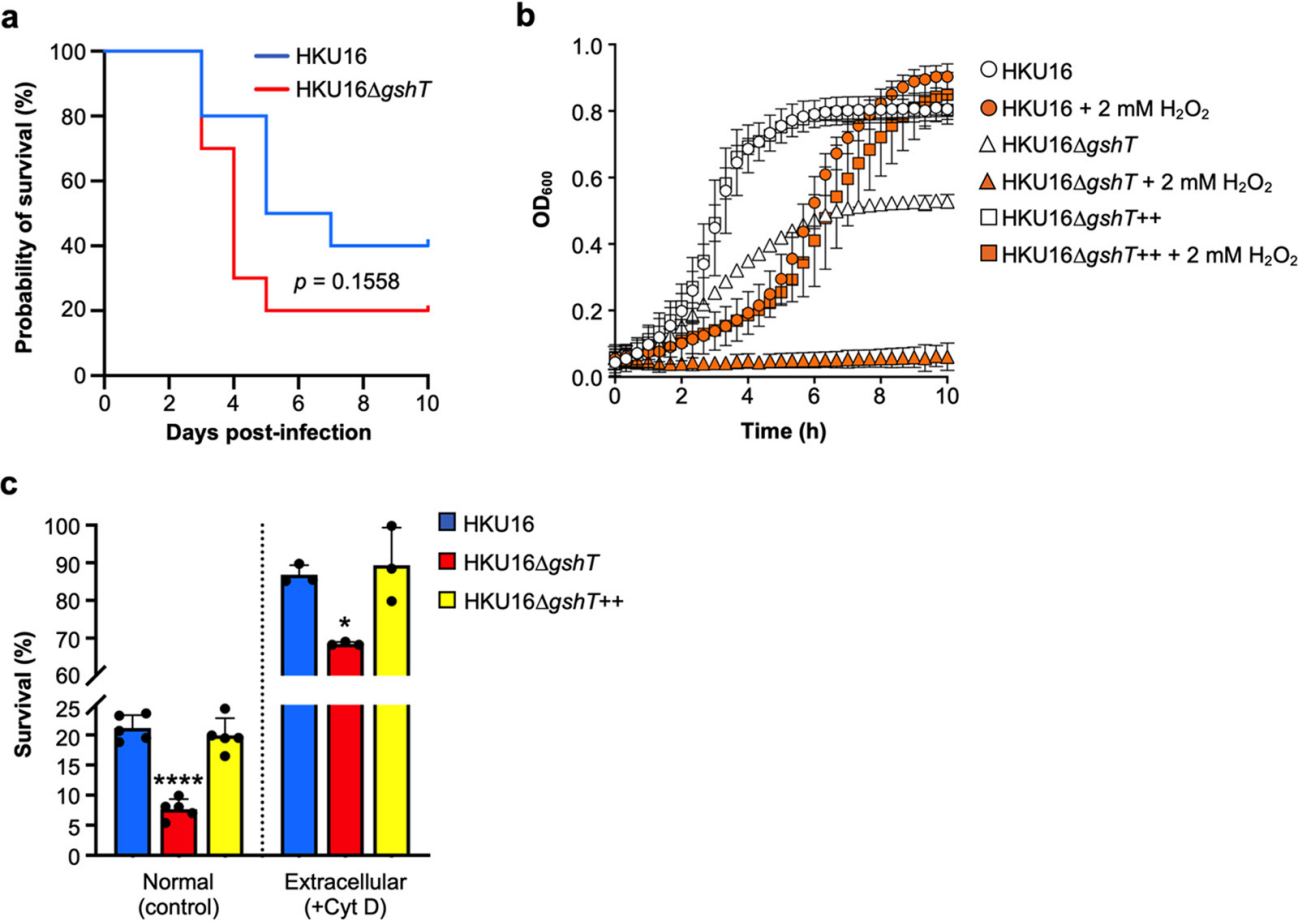
Glutathione regulates resistance to oxidative stress and killing by human neutrophils. (a) Survival of mice following intraperitoneal challenge. Groups of 10 C57BL/6J mice were challenged intraperitoneally with 1.1 × 10^7^ CFU of HKU16 wild type and 9.8 × 10^6^ CFU of HKU16Δ*gshT*. Survival of mice was monitored daily for 10 days. Data are presented as a Kaplan-Meier plot. (b) Growth curves of indicated HKU16 strains, with or without the addition of 2 mM hydrogen peroxide (H_2_O_2_) (*n* = 3). Data are presented as mean values ± SD. Growth curves of the wild-type and complemented mutant strain are very similar with substantial overlap. (c) Human neutrophil killing assay (normal, control) showing the percent survival of indicated strains following coculture with human neutrophils *in vitro* for 30 min at a multiplicity of infection of 0.1 (neutrophil/bacterial CFU) (*n *= 4). Cytochalasin D (Cyt D) is a potent inhibitor of actin polymerization, preventing phagocytosis and intracellular uptake of bacteria (extracellular, +Cyt D) (*n *= 3). Data are presented as mean values ± SD. Statistical significance was assessed using one-way ANOVA with Dunnett’s multiple-comparison *post hoc* test against the HKU16 wild-type control group (****, *P < *0.0001; *, *P < *0.05 for HKU16Δ*gshT*).

Given the altered virulence gene expression of HKU16Δ*gshT*, we investigated the virulence of both mutant and wild type in a mouse model of GAS invasive disease. Although there was a trend toward greater mortality with the mutant, loss of *gshT* had no significant effect on the pathogenesis of HKU16 following systemic challenge (Fig. 4a). Given that loss of *gshT* may play a role in niche-specific adaption and that pathogenicity in mice may not reflect infection in humans, we chose to further explore the role of GshT in resisting oxidative stress using *in vitro* and *ex vivo* models.

ROS are produced as a by-product of aerobic metabolism and are capable of damaging nucleic acid, protein, and cell membranes. The reduced aerobic growth of the *gshT* mutant strain suggests a role for glutathione in the resistance of GAS to H_2_O_2_-induced oxidative stress, providing the bacterium with the capacity to survive the millimolar concentrations of peroxide that it can produce (19, 44). We therefore analyzed the growth kinetics of HKU16Δ*gshT* in the presence of exogenous H_2_O_2_. Treatment with 2 mM H_2_O_2_ completely inhibited the growth of HKU16Δ*gshT*, while it only extended the lag phase of the HKU16 wild-type and complemented strains, which both reached bacterial densities equivalent to those of untreated cultures in the stationary phase (Fig. 4b). These results confirm that knockout of *gshT* renders GAS hypersensitive to oxidative stress.

ROS production by host neutrophils is a major defense mechanism against invading pathogens, crucial to the early control of infections (45–47). To determine the physiological role of glutathione assimilation during infection by GAS, we examined the susceptibility of the *gshT* mutant using an *ex vivo* human neutrophil model. As shown in Fig. 4c, knockout of *gshT* significantly reduced survival following exposure of GAS to human neutrophils compared to the HKU16 wild-type and complemented strain. To investigate the involvement of neutrophil phagocytosis, we made use of cytochalasin D, a potent inhibitor of actin polymerization and phagocytosis (48). In the presence of cytochalasin D, survival of the HKU16 wild-type and complemented strain was again greater than the survival of HKU16Δ*gshT* (Fig. 4c), demonstrating that glutathione import is critical for the ability of GAS to evade both intra- and extracellular killing by human neutrophils, either directly or indirectly by differential expression of virulence factors.

## DISCUSSION

GAS is an important human pathogen and a common cause of a wide variety of infections, ranging from mild illnesses such as pharyngitis and localized skin infections to more severe invasive disease, including sepsis, streptococcal toxic shock syndrome, and necrotizing fasciitis. Combined, these invasive infections and autoimmune sequelae triggered by GAS account for serious morbidity and mortality worldwide, causing an estimated annual burden of >500,000 deaths (49). Despite this immense burden of disease, there is currently no commercial vaccine available to prevent GAS infections (50).

GAS is a facultative anaerobic microorganism that relies on glycolysis and pyruvate metabolism for energy production. The upper respiratory tract and skin constitute the primary ecological niche of GAS (51). As a lactic acid bacterium, GAS is unable to synthesize heme and lacks catalase, a heme-containing peroxidase expressed by numerous other bacterial species to resist oxidative stress and survive in aerobic environments (52). To establish an infection, GAS must therefore rely on other strategies to persist and thrive in oxygen-rich tissues and to combat bactericidal ROS generated endogenously and by host innate immune cells (52). This study reveals the import of host-derived glutathione as a novel mechanism utilized by GAS to counter redox stress during aerobic metabolism and innate immune assault. Our findings establish GshT as a critical component of the glutathione import machinery and provide important insights into bacterial survival responses to glutathione depletion that are associated with extensive alterations in metabolic and virulence gene expression profiles in GAS.

There is little knowledge regarding the physiological consequences of glutathione starvation in bacteria such as GAS that lack both *de novo* glutathione biosynthesis and biosynthetic pathways for production of functionally analogous cysteine derivatives. Using knockout mutagenesis, we demonstrate that when starved for intracellular glutathione, GAS suffers from oxidative stress during aerobic metabolism. This triggers the enhanced production of the redox equivalent NADPH. This response to oxidative stress is also observed in eukaryotic cells (21, 22), suggesting a broadly conserved stress-response mechanism. However, GAS lacks the major bacterial pathways of cytosolic NADPH generation, the oxPPP, and the TCA cycle (23, 24). We therefore hypothesize that in GAS, excess NADPH is provided via increased glycolytic flux through GapN (26).

**FIG 5:**
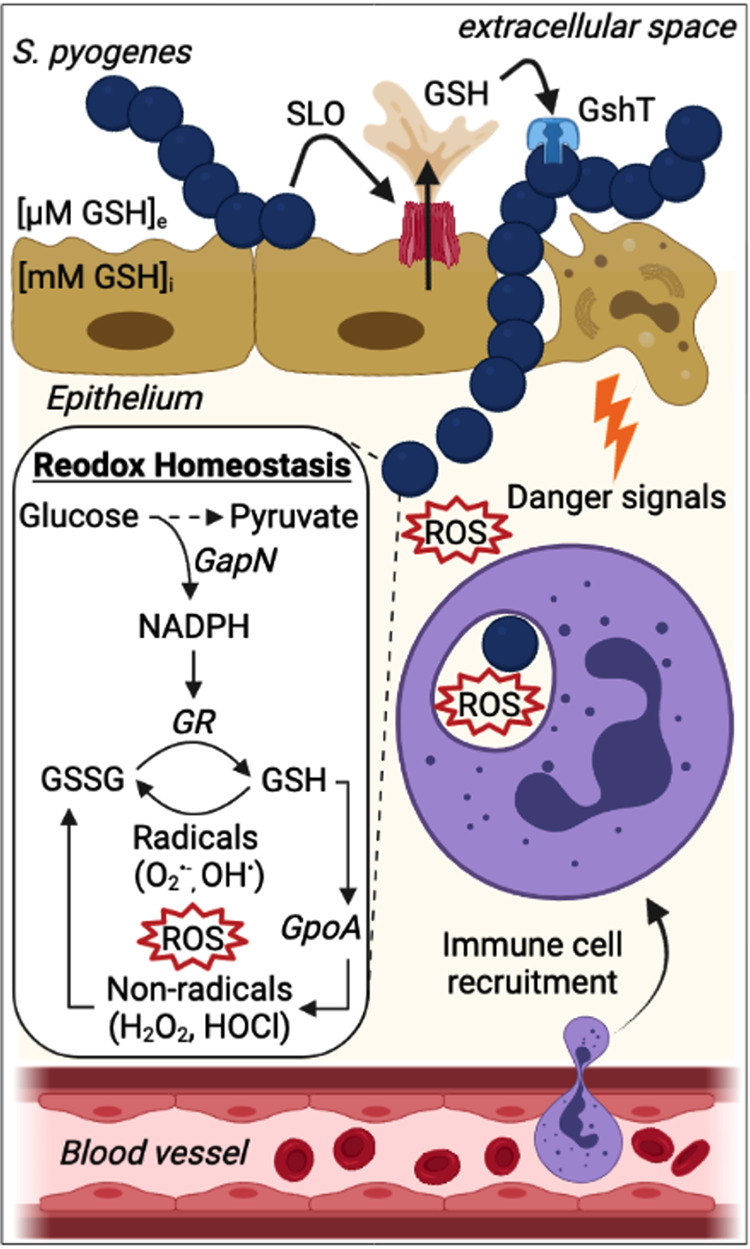
Role for glutathione in host colonization and innate immune evasion. After initial adherence to host epithelial cells, GAS secrete the pore-forming toxin SLO, which binds to host cell membranes and then oligomerizes to form large pores inducing the release of glutathione from perforated host cells due to the significant concentration gradient (~1,000-fold) across the plasma membrane of eukaryotic cells (16). GshT then facilitates the import of extracellular host-derived glutathione enabling aerobic growth of GAS and triggering niche adaptation associated with an altered gene expression profile. Damaged tissues and cells release danger signals to recruit innate immune cells such as neutrophils (87). Intracellular glutathione protects GAS from reactive oxygen species (ROS) produced from infiltrating immune cells in different ways, such as (i) GSH directly and nonenzymatically reduces radical forms of oxygen, while (ii) GSSG is predominantly produced by the enzymatic catalysis of hydroperoxides by the glutathione peroxidase GpoA (12, 88). Glutathione reductase (GR) restores intracellular levels of GSH by reducing GSSG using NADPH as an electron donor, thereby maintaining the cellular supply of GSH. This figure was created with BioRender.com.

The initial step in GAS pathogenesis is the colonization of the respiratory or skin epithelia (53). Once colonization is established, GAS may multiply extracellularly before penetrating deeper underlying tissues. The pore-forming toxin SLO is one of the key GAS virulence factors that promotes both host colonization and tissue injury (16, 54–56). We have recently demonstrated that epithelial cell damage by SLO results in a substantial release of host cytosolic glutathione stores into the extracellular environment, promoting bacterial growth (16). During the colonization stage of GAS infection, the host response is largely dependent on the antimicrobial activities of neutrophils that are rapidly recruited and engulf the invading bacteria at the site of infection (57). Our data support a model where SLO-mediated cytotoxicity provides a supply of an otherwise scarce extracellular antioxidant that equips GAS with higher tolerance to bactericidal ROS from infiltrating immune cells (Fig. 5). GAS interaction with human neutrophils is also associated with upregulation of GAS *gpoA*, responsible for the enzymatic catalysis of hydroperoxides (also referred to as *bsa*) (12, 58), underscoring the importance of glutathione acquisition during GAS infection.

Host-derived glutathione plays an important role in modifying bacterial fitness and virulence of facultative intracellular pathogens, where it can function as an allosteric activator for major regulatory proteins to increase the expression of virulence factors (31, 32, 34). Our study suggests that extracellular pathogens such as GAS may exploit host cytosolic glutathione stores to regulate niche adaptation. Loss of glutathione import and the resulting oxidative stress status during *in vitro* growth triggered a marked alteration in the transcriptome of GAS that selectively affected metabolic and virulence genes, including the *speB*-*prsA* operon. SpeB is a broad-spectrum cysteine protease that targets multiple host and bacterial proteins, including GAS surface and secreted proteins (59, 60), as well as proteins from other bacterial pathogens that occupy similar niches (61). While the precise role of SpeB in disease progression and pathogenesis remains to be fully established, a clinical correlation between invasive disease severity and diminished SpeB production has been reported in the highly invasive GAS M1T1 clone (62, 63). Thus, it is possible that decreased SpeB production by GAS *emm*12 HKU16Δ*gshT* results in increased virulence in a mouse model of systemic infection. It would be interesting in the future to extend this work to other murine models of GAS infection, such as a mouse model of skin and soft tissue infection, to assess the importance of glutathione import in GAS during earlier stages of infection that are characterized by extensive neutrophil infiltration (45–47).

In addition to acting as a cofactor, intracellular glutathione also protects catalytic cysteine residues from irreversible oxidation during oxidative stress through *S*-glutathionylation (64). This process can become crucial for preventing modification of the bacterial cysteine proteome during phagocytosis and host-pathogen interactions (65). In GAS, the quorum-sensing transcriptional regulator of the *speB*-*prsA* operon, RopB/Rgg, is unusually rich (a total of 10) in cysteine residues. Some of these cysteine residues are critical for RopB/Rgg activity and could thus serve as redox switches in oxidative stress sensing to regulate *speB* expression levels (66–69), providing a possible explanation for strongly repressed *speB* promoter activities in the HKU16Δ*gshT* mutant (Fig. 3b). Redox regulation of quorum-sensing regulators is an emerging regulatory mechanism in bacterial pathogens (70–72) which allows bacteria to sense and rapidly respond to changing redox conditions.

Collectively, our results offer fundamental insights into host adaptation pathways that enable extracellular bacterial pathogens to exploit the abundance of glutathione in the host cytosol as a signal to regulate niche adaption. This work inspires future investigation into understanding how glutathione shapes carbohydrate metabolism and virulence factor production in GAS.

## MATERIALS AND METHODS

### Bacterial strains and growth conditions.

The *emm12* GAS scarlet fever isolate HKU16 (18) and isogenic derivatives were routinely grown at 37°C on 5% horse blood agar, statically in Todd-Hewitt broth supplemented with 1% yeast extract (THY), or using chemically defined medium (16). Escherichia coli strain MC1061 was used for cloning and was grown in Luria-Bertani medium (LB). Where required, spectinomycin was used at 100 μg mL^−1^ (both GAS and E. coli). Bacterial strains and plasmids used in this study are listed in Table S2 in the supplemental material. Kinetic measurements of bacterial growth were performed in 96-well microtiter plates using the FLUOstar Omega microplate reader (BMG Labtech) at 37°C. GAS strains were first grown overnight on horse blood agar and then inoculated into THY medium to an optical density at 600 nm (OD_600_) of 0.1, using six technical replicates per strain and growth condition. Plates were gently shaken at 400 rpm for 20 s before each measurement. Where indicated, reducing agents were freshly prepared as a 100× stock solution in the respective growth medium and added to cultures prior to growth measurements.

10.1128/mbio.00676-22.6 TABLE S2 List of bacterial strains, plasmids, and primers used in this study. Download Table S2, DOCX file, 0.02 MB. Copyright © 2022 Brouwer et al. 2022 Brouwer et al. https://creativecommons.org/licenses/by/4.0/ This content is distributed under the terms of the Creative Commons Attribution 4.0 International license.

### *HKU16*Δ*gshT* isogenic mutant construction.

A markerless HKU16Δ*gshT* isogenic mutant strain was generated using a highly efficient plasmid (pLZts) as previously described (73). PCR primers were designed to keep the first and last 24 nucleotides (8 amino acids), respectively, of the *gshT* coding sequence intact to reduce the possibility of downstream effects. The same protocol was used to reintroduce the HKU16 *gshT* wild-type gene back into HKU16Δ*gshT* to generate the complemented strain. All PCR primer sequences are provided in Table S2. Gene deletion and complementation were confirmed by DNA sequence analysis (Genetic Research Services, University of Queensland, Brisbane, Australia).

### Scanning electron microscopy.

Scanning electron microscopy (SEM) studies were undertaken at the Centre for Microscopy and Microanalysis at the University of Queensland. GAS strains HKU16 and HKU16Δ*gshT* were grown overnight in THY at 37°C. Bacteria were washed twice with phosphate-buffered saline (PBS) preceding glutaraldehyde fixation. Samples were then dehydrated and assisted with a Pelco biowave regimen via a series of ethanol treatments (30 to 100% EtOH), one treatment with 100% EtOH/hexamethyldisilazane (HMDS; 1:1) and, finally, two treatments with 100% HMDS. Samples were applied to coverslips coated with poly-l-lysine (1 mg/mL) before being air-dried for 2 h. Coverslips were attached to 13-mm SEM stubs with double-sided carbon tabs, plasma cleaned for 10 min in an Evactron decontaminator (XEI Scientific), and coated with two layers of platinum (first layer, 0° angle from above; second layer, 45° angle from above) using a turbomolecular pumped coater (Quorum Tech) following the manufacturer’s instructions. Samples were imaged in a Jeol JSM 7100F or Jeol JSM 7800F field emission SEM at an accelerating voltage of 1 to 3 kV and at a `working distance of 10 mm.

### Intracellular metabolite quantification.

Samples were prepared either from overnight growth at 37°C on 5% horse blood agar or from THY cultures. Briefly, 25 mL of THY was inoculated with GAS HKU16 to an OD_600_ of 0.1 and grown to the desired growth phase at 37°C. Ten milliliters of culture per OD_600_ of 0.5 were then transferred to a new tube and centrifuged for 10 min at 8,000 × *g* at 4°C. The bacterial pellet was resuspended 1 mL of ice-cold PBS and centrifuged for 2.5 min at 8,000 × *g* at 4°C. Finally, the bacterial pellet was resuspended in 0.5 mL of ice-cold PBS and transferred to a prechilled Lysing Matrix B tube (MP Biomedicals; catalog no. MP116911050). Bacterial cells were lysed using the FastPrep-2 5G bead beating grinder and lysis system (MP Biomedicals) with 3 cycles of 40 s at a speed setting of 9.0 with 180-s-break intervals. Lysates were centrifuged for 2 min at 16,000 × *g* at 4°C. The clear supernatant was either used directly (horse blood agar [HBA] plates) or after a freeze-thaw cycle (THY cultures) to determine intracellular concentrations of glutathione (oxidized GSSG/reduced GSH), NAD (NAD^+^/reduced NADH), NADP (oxidized NADP^+^/reduced NADPH) redox couples using GSH-Glo (Promega; catalog no. V6912), NAD/NADH-Glo (Promega; catalog no. G9071) and NADP/NADPH-Glo (Promega; catalog no. G9081) assays as per the manufacturer’s instructions. To determine total GSH (the sum of GSSG and GSH) levels, samples were mixed 1:1 with 2 mM TCEP prior to use. All assays were performed in white 96-well plates (Greiner Bio-one; catalog no. 655083). Luminescent intensity was measured using a FLUOstar Omega microplate reader. Sample readings were analyzed by Prism 9 software and normalized to the protein content of each sample, which was assessed using the Pierce bicinchoninic acid (BCA) protein assay kit (Pierce; catalog no. 23225).

### RNA isolation.

Total RNA was routinely isolated from bacterial cells using the RNeasy minikit (Qiagen; catalog no. 74106) as previously described (16). Briefly, GAS HKU16 was grown for 5 h to late logarithmic growth phase in THY medium. Two volumes of RNAprotect (Qiagen; catalog no. 76506) were added to the cultures, and bacterial cells were collected by centrifugation at 5,000 × *g* for 25 min at 4°C. RNA was isolated from dry pellets as per the manufacturer’s instructions with an additional mechanical lysis step using Lysing Matrix B tubes on the FastPrep-2 5G bead beating grinder and lysis system (MP Biomedicals). To ensure complete removal of contaminating DNA, RNA samples were further purified using the Turbo DNA-free kit (Invitrogen; catalog no. AM1907) according to the manufacturer’s instructions.

### RNA-seq analysis.

RNA-seq analysis was performed at the Australian Centre for Ecogenomics (University of Queensland, Brisbane, Australia). cDNA libraries were prepared from total RNA using TruSeq stranded total RNA library prep with Ribo-Zero Plus rRNA depletion kit (Illumina). Sequencing of the cDNA libraries was performed on the NovaSeq 6000 system (Illumina) on a 2 × 150-bp SP flow cell run generating an average of 20 million reads per sample. The quality of raw reads was assessed using FastQC v.0.11.0 (74). Reads <45 bp were filtered using cutadapt v.2.8 (75), and rRNA was filtered using SortMeRNA v.4.2.0 (76) with a database of rRNA from GAS strains 5448, SF370, and HKU488. Reads were aligned to the HKU16 reference genome (GCF_000275625.1) using BWA-MEM from bwa v.0.7.17 (77). Fragment counting (-p) was performed using featureCounts from subreads v. 2.0.0 (78) in a strand-specific fashion (-s 2), counting multimapped reads in the feature with largest overlap of the read (-O –largestOverlap). Differential expression of features was calculated using DEseq2 v.1.32.0 (79) and edgeR v.3.34.1 (80) in R v.4.1.1. Results were filtered to have a base mean expression of >100 fragments.

### Quantitative real-time PCR.

For quantitative real-time PCR analysis, total RNA was converted to cDNA using the GoScript reverse transcription system (Promega; catalog no. A5001). The resulting cDNA libraries were used to assess relative gene expression as previously described (16). Briefly, quantitative real-time PCR was performed on selected genes using the primers specified in Table S2, using SYBR green master mix (Applied Biosystems) according to the manufacturer’s instructions. All data were analyzed using QuantStudio real-time PCR software v1.1 (QuantStudio 6 Flex; Life Technologies). Relative gene expression was calculated using the threshold cycle (2^−ΔΔ^*^CT^*) method with *gyrA* as the reference housekeeping gene (81). All experiments were performed in biological triplicates and measured in technical triplicates.

### Immunoblot analysis.

GAS HKU16 strains were grown to late logarithmic growth phase in THY. Filter-sterilized culture supernatants were precipitated with 10% trichloroacetic acid (TCA). TCA precipitates were resuspended in a loading buffer (normalized to OD_600_ of cultures) in the presence of 100 mM DTT. Samples were boiled for 10 min, subjected to SDS-PAGE, and then transferred to a polyvinylidene difluoride membrane for detection of immunoreactive bands using a LI-COR Odyssey imaging system (LI-COR Biosciences). For detection of SpeB, affinity-purified rabbit antibody to SpeB (catalog no. PBI222; Toxin Technology) was used at a 1:1,000 dilution. Anti-rabbit IgG (H+L) (DyLight 800 4× polyethylene glycol [PEG] conjugate; NEB; catalog no. 5151P) was used as the secondary antibody (1:10,000).

### Murine intraperitoneal infection model.

Systemic infection of mice was established by intraperitoneal injection of GAS HKU16 prepared from a frozen stock as previously described (82). Briefly, bacterial strains were cultured to late logarithmic growth phase in THY at 37°C. Cells were washed twice using ice-cold THY, resuspended in ice-cold THY supplemented with 15% (vol/vol) glycerol, aliquoted, snap frozen in liquid nitrogen, and stored at −80°C. Prior to infection, aliquots were thawed, washed twice in ice-cold PBS, and adjusted to a dose of 10^7^ CFU in a volume of 100 μL PBS. The infection dose was injected into sex- and age-matched (6- to 8-week-old) C57BL/6J mice (*n* = 10 for all groups). Mice were housed in groups of five with free access to food and water throughout the experiment. Survival of infected mice was monitored daily for a period of 10 days.

### *Ex vivo* human neutrophil model.

Human neutrophil killing assays were performed as previously described (83). Briefly, human neutrophils were isolated from fresh heparinized whole blood using PolymorphPrep density gradient centrifugation (Axis-Shield) as per the manufacturer’s instructions. Hypotonic lysis was performed to remove residual erythrocytes. Purified neutrophils were infected with GAS at a multiplicity of infection of 0.1 (1 × 10^6^ cells mL^−1^ neutrophils/1 × 10^5^ bacterial CFU mL^−1^) in RPMI 1640 medium with 2% heat-inactivated serum, centrifuged for 5 min at 370 × *g* to synchronize phagocytosis, and then incubated for 30 min at 37°C under 5% CO_2_. Internal control wells without neutrophils were used to determine baseline bacterial counts at the assay endpoint. The bacterial infection dose was prepared from cultures grown to mid-logarithmic growth phase in THY supplemented with 2 mM l-cysteine to synchronize bacterial growth rates. To prevent phagocytosis, the neutrophil killing assay was performed with the addition of 10 μg mL^−1^ cytochalasin D (Sigma). Infected neutrophils were lysed using 0.025% Triton X-100 and serially diluted in sterile Milli-Q water and then plated on THY agar for bacterial enumeration.

### Statistical analysis.

All statistical analysis was completed using Prism software (GraphPad; version 9.2.0). Significance was calculated using one-way analysis of variance (ANOVA) with Dunnett’s multiple comparisons *post hoc* test. A *P* value of less than 0.05 was determined to be statistically significant.

### Ethics statement.

Human blood donation for use in neutrophil killing assays and NET degradation assays were conducted in accordance with the *Australian National Statement on Ethical Conduct in Human Research (2007)* (84) in compliance with the regulations governing experimentation on humans and was approved by the University of Queensland medical research ethics committee (2010001586). Informed consent was obtained from all participants. Animal experiments were performed according to the *Australian code of practice for the care and use of animals for scientific purposes*. Permission was obtained from the University of Queensland ethics committee to undertake this work (SCMB/140/16/NHMRC). Animal holding rooms were held at 22°C (with a range of 20 to 26°C). Humidity was kept between ~50 and 70%. A 12-h light/dark cycle (6 a.m. to 6 p.m.) was used. Temperature and light cycle were both monitored. Humidity and temperature were recorded daily by animal holding room technicians.

### Data availability.

The RNA-seq data discussed in this publication have been deposited in NCBI's Gene Expression Omnibus (85) and are accessible through GEO Series accession number GSE198061 (https://www.ncbi.nlm.nih.gov/geo/query/acc.cgi?acc=GSE198061).
