# Supplementary figures and images for: Streptococcus pyogenes Hijacks Host Glutathione for Growth and Innate Immune Evasion
Source: mBio. 2022 Apr 25;13(3):e00676-22. doi: 10.1128/mbio.00676-22 (PMC9239160; doi:10.1128/mbio.00676-22)

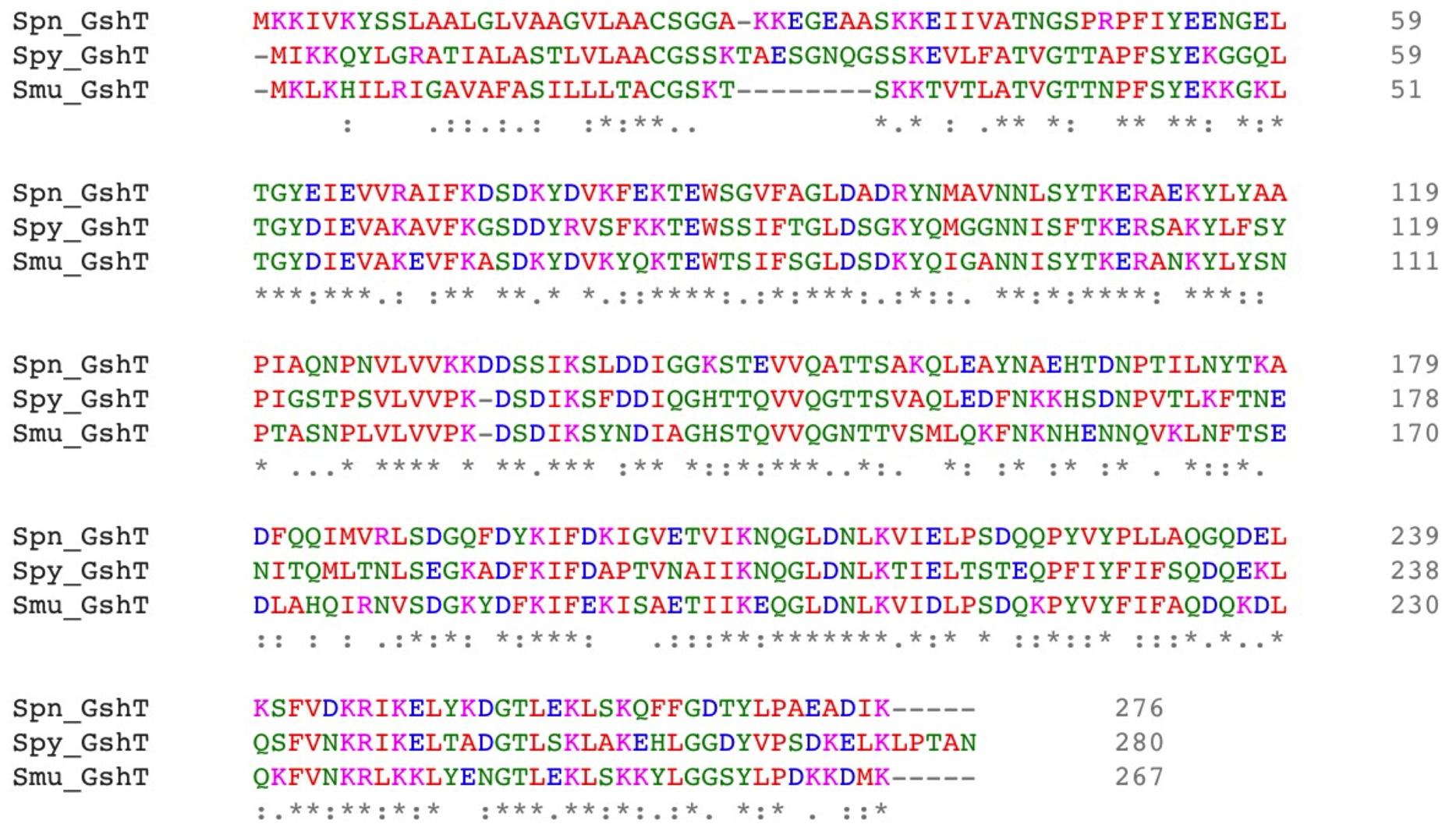

Supplement: FIG S1 [file mbio.00676-22-sf001.tif]

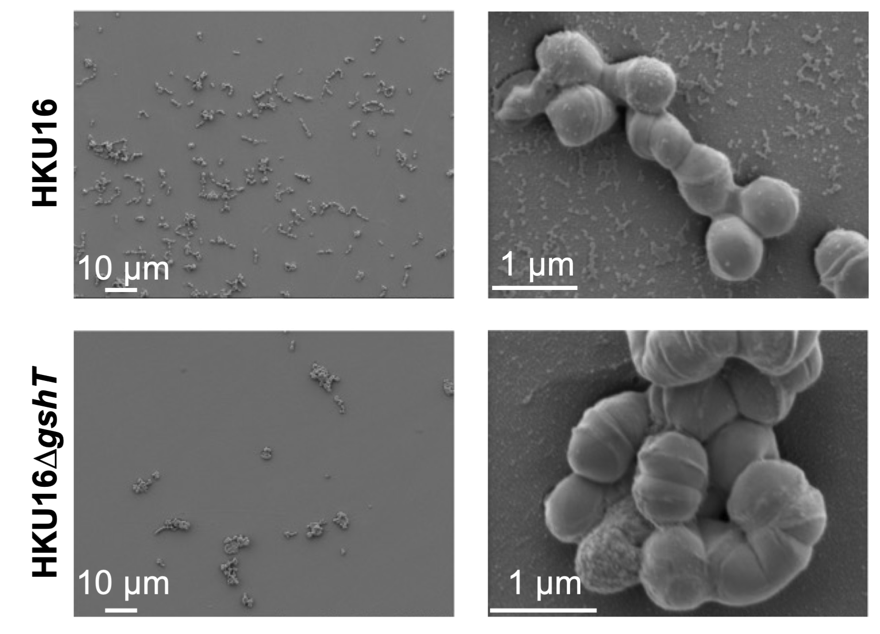

Supplement: FIG S2 [file mbio.00676-22-sf002.tif]

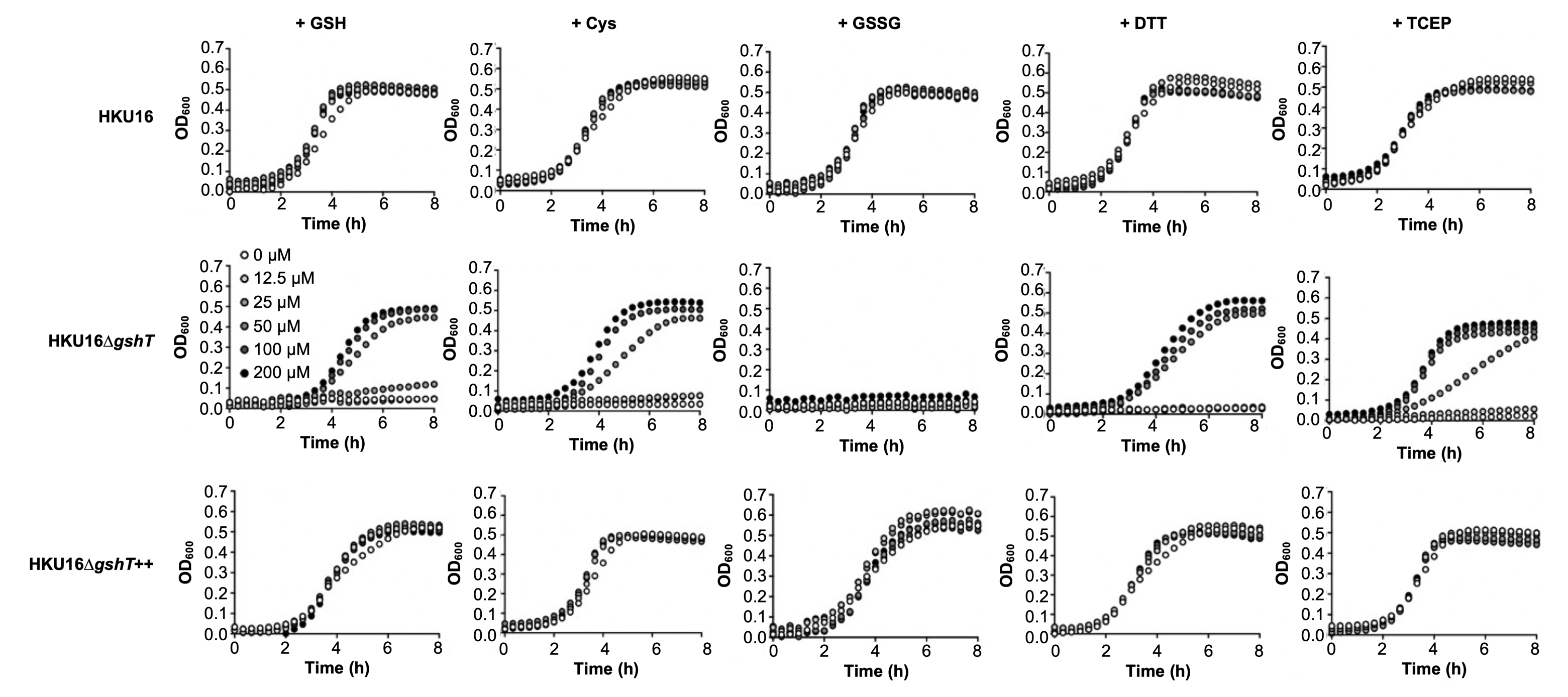

Supplement: FIG S3 [file mbio.00676-22-sf003.tif]

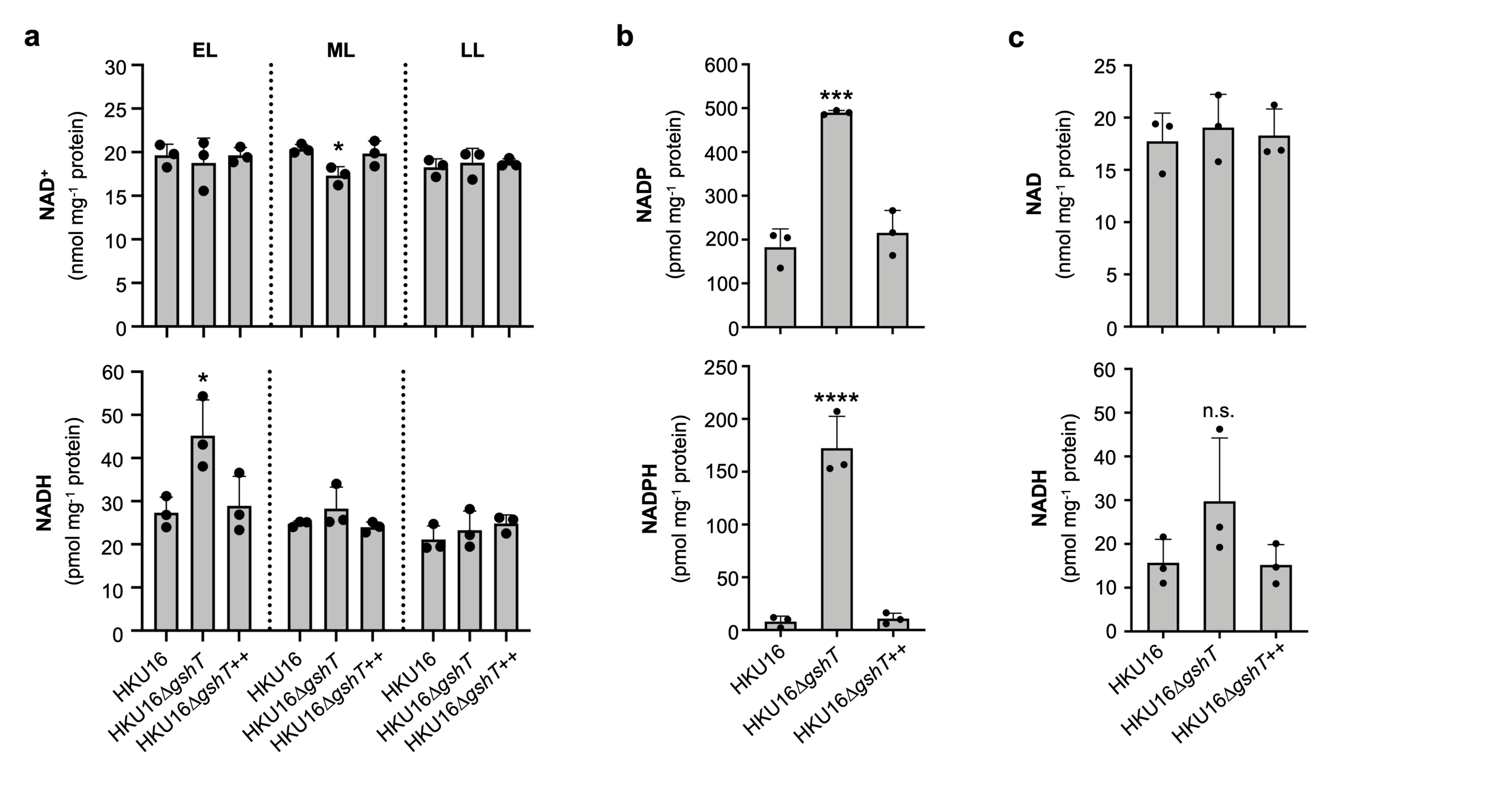

Supplement: FIG S4 [file mbio.00676-22-sf004.tif]
