# Supplementary material for: Streptococcus pyogenes Hijacks Host Glutathione for Growth and Innate Immune Evasion
Source: mBio. 2022 Apr 25;13(3):e00676-22. doi: 10.1128/mbio.00676-22 (PMC9239160; doi:10.1128/mbio.00676-22)
Supplement: TABLE S1 [file mbio.00676-22-st001.docx]

| **Gene_id** | **Gene** | **Product** | **log2FC** | ***p*adj** |
| --- | --- | --- | --- | --- |
| RS02915 | *fruR* | DeoR/GlpR transcriptional regulator | 1.9 | 1.1E-19 |
| RS02920 | *fruB* | 1-phosphofructokinase | 1.9 | 4.3E-20 |
| RS03585 | *-* | ABC transporter ATP-binding protein | 1.9 | 1.8E-30 |
| RS03580 | *-* | ABC transporter permease | 1.8 | 6.3E-39 |
| RS02030 | *-* | PRD domain-containing protein | 1.7 | 1.5E-07 |
| RS03860 | *lanC* | Lanthionine synthetase C family protein | 1.6 | 7.7E-12 |
| RS00215 | *-* | Hypothetical protein | 1.6 | 5.6E-21 |
| RS03575 | *-* | ABC transporter substrate-binding protein | 1.5 | 1.5E-28 |
| RS02685 | *-* | Lipopolysaccharide biosynthesis protein | 1.5 | 7.6E-14 |
| RS02680 | *-* | DUF2304 domain-containing protein | 1.5 | 1.2E-15 |
| RS03855 | *srtT* | ABC transporter ATP-binding protein | 1.4 | 1.2E-20 |
| RS02690 | *-* | DUF2142 domain-containing protein | 1.4 | 1.3E-13 |
| RS07255 | *-* | DUF1492 domain-containing protein | 1.4 | 1.4E-08 |
| RS08340 | *slo* | Cholesterol-dependent cytolysin | 1.4 | 3.4E-05 |
| RS02925 | *fruA* | PTS sugar transporter subunit IIA | 1.4 | 3.9E-11 |
| RS03895 | *-* | Helix-turn-helix transcriptional regulator | 1.3 | 6.1E-06 |
| RS05170 | *grab* | G-related a2-macroglobulin-binding protein | 1.3 | 9.4E-15 |
| RS08345 | *ifs* | NAD glycohydrolase inhibitor | 1.3 | 2.8E-04 |
| RS00125 | *-* | Lytic transglycosylase protein | 1.3 | 3.0E-11 |
| RS02675 | *-* | Glycosyltransferase family 2 protein | 1.3 | 8.1E-12 |
| RS03865 | *-* | Lantibiotic dehydratase | 1.2 | 2.5E-11 |
| RS06020 | *cysK* | Cysteine synthase A | 1.2 | 9.6E-16 |
| RS01665 | *-* | YozE family protein | 1.2 | 4.1E-15 |
| RS02935 | *-* | Glycoside hydrolase family 73 protein | 1.2 | 2.5E-17 |
| RS04210 | *-* | DUF1836 domain-containing protein | 1.2 | 9.1E-15 |
| RS00220 | *-* | DNA translocase FtsK | 1.2 | 7.4E-12 |
| RS04205 | *-* | Hemolysin III family protein | 1.1 | 4.5E-14 |
| RS01670 | *lysM* | Peptidoglycan-binding protein | 1.1 | 1.8E-29 |
| RS00225 | *-* | Helix-turn-helix transcriptional regulator | 1.1 | 1.3E-11 |
| RS01175 | *sstT* | Serine/threonine transporter SstT | 1.1 | 2.5E-13 |
| RS06900 | *pncA* | Nicotinamidase | 1.1 | 8.1E-05 |
| RS00230 | *cadX* | Cd/Zn-sensing transcriptional regulator | 1.1 | 2.5E-07 |
| RS02415 | *-* | YueI family protein | 1.1 | 1.3E-06 |
| RS08270 | *-* | ABC transporter permease | 1.1 | 6.3E-15 |
| RS02670 | *-* | Sulfatase-like hydrolase/transferase | 1.1 | 3.4E-09 |
| RS02035 | *-* | PTS beta-glucoside transporter subunit IIBCA | 1.1 | 8.5E-04 |
| RS01060 | *-* | ABC transporter ATP-binding protein | 1.1 | 1.7E-16 |
| RS06010 | *comFA* | DEAD/DEAH box helicase | 1.0 | 2.1E-04 |
| RS09170 | *-* | Hypothetical protein | 1.0 | 4.9E-03 |
| RS04850 | *-* | Recombinase family protein | 1.0 | 1.4E-10 |
| RS01885 | *-* | AMP-binding protein | 1.0 | 7.2E-13 |
| RS0109275 | *-* | PepSY domain-containing protein | 1.0 | 1.9E-07 |
| RS00495 | *groES* | Chaperone | -1.0 | 1.3E-02 |
| RS08425 | *-* | PTS fructose transporter subunit IIC | -1.0 | 1.1E-04 |
| RS04580 | *amaP* | Alkaline shock response protein | -1.0 | 6.6E-21 |
| RS04570 | *-* | Envelope stress response protein | -1.0 | 1.3E-17 |
| RS02310 | *-* | (S)-acetoin forming diacetyl reductase | -1.0 | 4.1E-06 |
| RS00005 | *-* | ParB/RepB/Spo0J family partition protein | -1.0 | 1.7E-08 |
| RS04655 | *-* | GntR family transcriptional regulator | -1.0 | 1.3E-20 |
| RS00695 | *emm* | M protein | -1.0 | 1.0E-02 |
| RS01860 | *-* | ATP-binding cassette protein | -1.0 | 1.3E-03 |
| RS05870 | *-* | YesL family protein | -1.0 | 1.4E-05 |
| RS05865 | *-* | Sensor histidine kinase | -1.1 | 9.7E-06 |
| RS06385 | *copA* | Copper-translocating P-type ATPase | -1.1 | 9.0E-06 |
| RS00195 | *-* | DUF1700 domain-containing protein | -1.1 | 4.2E-04 |
| RS06840 | *dnaJ* | Molecular chaperone | -1.1 | 1.1E-02 |
| RS02855 | *-* | Glycerophosphoryl diester phosphodiesterase | -1.1 | 1.3E-28 |
| RS00500 | *groEL* | Chaperone | -1.1 | 1.2E-05 |
| RS08430 | *-* | RidA family protein | -1.1 | 3.7E-05 |
| RS02845 | *-* | ABC transporter ATP-binding protein | -1.1 | 1.3E-21 |
| RS00010 | *-* | Trypsin-like peptidase | -1.1 | 4.6E-08 |
| RS07930 | *-* | transaldolase | -1.1 | 6.9E-03 |
| RS00510 | *-* | C69 family dipeptidase | -1.1 | 9.3E-14 |
| RS00460 | *-* | Imidazolonepropionase | -1.1 | 2.2E-04 |
| RS00575 | *-* | PTS sugar transporter subunit IIC | -1.1 | 1.8E-02 |
| RS00135 | *sdaAB* | L-serine ammonia-lyase subunit beta | -1.2 | 2.6E-14 |
| RS04560 | *-* | Envelope stress response protein | -1.2 | 1.5E-37 |
| RS03125 | *-* | Orotate phosphoribosyltransferase | -1.2 | 1.4E-03 |
| RS00670 | *-* | Response regulator transcription factor | -1.2 | 5.5E-05 |
| RS08175 | *lmb* | Laminin-binding adhesin | -1.2 | 3.5E-06 |
| RS08015 | *-* | Pullulanase | -1.2 | 5.4E-14 |
| RS02840 | *-* | Efflux RND transporter periplasmic adaptor | -1.2 | 1.6E-16 |
| RS02830 | *carA* | Carbamoyl-phosphate synthase | -1.2 | 7.4E-09 |
| RS00865 | *-* | N-acetylmannosamine-6-phosphate 2-epimerase | -1.2 | 5.3E-03 |
| RS04795 | *-* | Glycoside hydrolase family 1 protein | -1.2 | 3.2E-03 |
| RS00350 | *nrdD* | Anaerobic ribonucleoside-triphosphate reductase | -1.2 | 2.9E-12 |
| RS02850 | *-* | ABC transporter permease | -1.2 | 9.5E-26 |
| RS07515 | *-* | MFS transporter | -1.3 | 9.4E-05 |
| RS02825 | *-* | Aspartate carbamoyltransferase catalytic subunit | -1.3 | 2.1E-10 |
| RS00820 | *-* | TIGR00266 family protein | -1.4 | 1.1E-16 |
| RS03120 | *pyrF* | Orotidine-5'-phosphate decarboxylase | -1.4 | 3.3E-04 |
| RS04105 | *-* | Purine permease | -1.4 | 8.3E-15 |
| RS02240 | *-* | N-Acetylgalactosamine EIIC | -1.4 | 3.2E-03 |
| RS02815 | *pyrR* | Bifunctional pyr operon transcriptional regulator | -1.4 | 1.9E-06 |
| RS00665 | *-* | ABC transporter permease | -1.4 | 7.7E-04 |
| RS08950 | *purD* | Phosphoribosylamine--glycine ligase | -1.4 | 7.0E-03 |
| RS02880 | *-* | Cation transporter | -1.4 | 8.8E-05 |
| RS02820 | *-* | Uracil permease | -1.4 | 7.6E-09 |
| RS00650 | *-* | Hypothetical protein | -1.5 | 8.6E-03 |
| RS08895 | *adhP* | Alcohol dehydrogenase AdhP | -1.5 | 6.6E-06 |
| RS08945 | *purE* | 5-(carboxyamino)imidazole ribonucleotide mutase | -1.5 | 1.2E-03 |
| RS07935 | *-* | PTS ascorbate transporter subunit IIC | -1.5 | 2.4E-06 |
| RS04100 | *-* | Xanthine phosphoribosyltransferase | -1.5 | 8.9E-15 |
| RS08025 | *ugpC* | Sn-glycerol-3-phosphate transporter protein | -1.5 | 3.9E-22 |
| RS00130 | *sdaAA* | L-serine ammonia-lyase subunit alpha | -1.5 | 1.5E-21 |
| RS03750 | *ptsA* | PTS sugar transporter subunit IIA | -1.6 | 1.7E-07 |
| RS04695 | *-* | Extracellular solute-binding protein | -1.6 | 2.5E-27 |
| RS03075 | *-* | ATP-dependent Clp protease ATP-binding subunit | -1.7 | 2.5E-02 |
| RS05315 | *-* | OFA family MFS transporter | -1.8 | 1.6E-12 |
| RS05905 | *-* | Hypothetical protein | -1.8 | 2.0E-22 |
| RS08395 | *-* | V-type ATP synthase subunit F | -1.9 | 7.0E-06 |
| RS08020 | *-* | Alpha-glucosidase | -1.9 | 1.1E-33 |
| RS08390 | *-* | V-type ATP synthase subunit A | -1.9 | 1.6E-16 |
| RS08380 | *-* | V-type ATP synthase subunit D | -1.9 | 1.8E-09 |
| RS08980 | *purS* | Phosphoribosylformylglycinamidine synthase | -1.9 | 7.4E-05 |
| RS00660 | *-* | ABC transporter ATP-binding protein | -2.0 | 2.0E-04 |
| RS00600 | *sdaB* | Streptodornase B | -2.0 | 6.1E-16 |
| RS03755 | *ptsB* | PTS sugar transporter subunit IIB | -2.0 | 6.1E-16 |
| RS07945 | *-* | BglG family transcription antiterminator | -2.0 | 3.1E-07 |
| RS06855 | *hrcA* | Heat-inducible transcriptional repressor | -2.1 | 5.5E-05 |
| RS08405 | *-* | Hypothetical protein | -2.1 | 8.2E-14 |
| RS07790 | *salT* | ABC transporter ATP-binding protein | -2.1 | 1.5E-04 |
| RS08400 | *-* | V-type ATPase subunit | -2.1 | 3.0E-13 |
| RS00655 | *-* | Efflux RND transporter periplasmic adaptor subunit | -2.1 | 1.0E-05 |
| RS08065 | *-* | PTS sugar transporter subunit EIIC | -2.1 | 3.1E-29 |
| RS05900 | *-* | Beta-N-acetylglucosaminidase | -2.1 | 5.8E-29 |
| RS03765 | *ptsD* | PTS sugar transporter subunit IID | -2.2 | 2.8E-17 |
| RS05910 | *-* | Glycoside hydrolase family 125 protein | -2.2 | 2.2E-25 |
| RS06850 | *grpE* | Nucleotide exchange factor GrpE | -2.2 | 2.0E-05 |
| RS02990 | *-* | 5'-nucleotidase | -2.2 | 5.2E-09 |
| RS08060 | *-* | endo-/exonuclease/phosphatase family protein | -2.2 | 2.9E-10 |
| RS05915 | *-* | Alpha-mannosidase | -2.2 | 1.3E-28 |
| RS06845 | *dnaK* | Molecular chaperone | -2.2 | 9.6E-07 |
| RS08385 | *-* | V-type ATP synthase subunit B | -2.2 | 6.3E-27 |
| RS00895 | *-* | ROK family protein | -2.2 | 6.9E-09 |
| RS00890 | *-* | Dihydrodipicolinate synthase family protein | -2.3 | 8.8E-08 |
| RS03760 | *ptsC* | PTS sugar transporter subunit IIC | -2.3 | 1.8E-20 |
| RS00875 | *-* | Sugar ABC transporter permease | -2.3 | 1.6E-09 |
| RS01490 | *lctO* | L-lactate oxidase | -2.3 | 1.2E-03 |
| RS00870 | *-* | ABC transporter substrate-binding protein | -2.4 | 8.9E-09 |
| RS05895 | *-* | Glycoside hydrolase family 1 protein | -2.4 | 3.5E-34 |
| RS08410 | *-* | V-type ATP synthase subunit K | -2.4 | 1.0E-07 |
| RS08985 | *purC* | Phosphoribosylaminoimidazolesuccinocarboxamide synthase | -2.5 | 7.6E-06 |
| RS00880 | *-* | Carbohydrate ABC transporter permease | -2.6 | 1.4E-12 |
| RS05710 | *arcA* | Arginine deiminase | -2.7 | 2.1E-21 |
| RS07835 | *lacA.2* | Galactose-6-phosphate isomerase subunit | -2.7 | 3.9E-05 |
| RS05885 | *-* | Sugar ABC transporter permease | -2.7 | 9.8E-19 |
| RS05875 | *-* | Extracellular solute-binding protein | -2.8 | 1.9E-21 |
| RS05880 | *-* | Carbohydrate ABC transporter permease | -2.8 | 2.1E-18 |
| RS08415 | *-* | V-type ATP synthase subunit I | -2.8 | 9.9E-17 |
| RS00885 | *-* | DUF624 domain-containing protein | -2.8 | 3.1E-09 |
| RS05700 | *argF* | Ornithine carbamoyltransferase | -2.8 | 1.9E-26 |
| RS05690 | *-* | Dipeptidase | -2.9 | 3.1E-14 |
| RS05685 | *arcC* | Carbamate kinase | -3.0 | 1.8E-22 |
| RS05695 | *arcD* | Arginine/ornithine antiporter | -3.0 | 1.8E-28 |
| RS01495 | *spyCEP* | CXC chemokine-degrading serine protease | -3.1 | 1.1E-07 |
| RS07825 | *lacC.2* | Tagatose-6-phosphate kinase | -3.1 | 1.2E-04 |
| RS07830 | *lacB.2* | Galactose-6-phosphate isomerase subunit | -3.4 | 1.2E-07 |
| RS00620 | *prsA* | Peptidylprolyl isomerase | -3.4 | 1.3E-20 |
| RS07795 | *salM* | DUF4135 domain-containing protein | -3.5 | 1.8E-10 |
| RS06370 | *-* | PTS sugar transporter subunit IIA | -3.7 | 1.6E-11 |
| RS07820 | *lacD.2* | Tagatose-bisphosphate aldolase | -3.9 | 4.7E-07 |
| RS07815 | *lacF* | PTS lactose/cellobiose transporter subunit IIA | -4.3 | 5.9E-13 |
| RS07810 | *lacE* | PTS transporter subunit EIIC | -4.4 | 2.0E-10 |
| RS06365 | *ptsIIB* | PTS sugar transporter subunit IIB | -4.7 | 1.4E-19 |
| RS06355 | *lacA.1* | Galactose-6-phosphate isomerase subunit | -4.8 | 1.0E-26 |
| RS07805 | *lacG* | 6-phospho-beta-galactosidase | -4.9 | 7.2E-12 |
| RS07800 | *-* | Type A2 lantipeptide | -5.1 | 9.6E-16 |
| RS06340 | *lacD.1* | Tagatose-bisphosphate aldolase | -5.2 | 1.5E-30 |
| RS06360 | *ptsIIC* | PTS galactitol transporter subunit IIC | -5.2 | 3.7E-30 |
| RS06350 | *lacB.1* | Galactose-6-phosphate isomerase | -5.5 | 1.9E-35 |
| RS01150 | *gshT* | ABC transporter substrate-binding protein | -6.9 | 3.8E-269 |
| RS00610 | *speB* | Cysteine protease | -10.7 | 1.7E-36 |
| RS00615 | *spi* | SpeB inhibitor | -10.7 | 1.7E-45 |
| RS09795 | *orf-3/speB* | Hypothetical protein | -11.1 | 8.8E-34 |
