# Supplementary material for: Streptococcus pyogenes Hijacks Host Glutathione for Growth and Innate Immune Evasion
Source: mBio. 2022 Apr 25;13(3):e00676-22. doi: 10.1128/mbio.00676-22 (PMC9239160; doi:10.1128/mbio.00676-22)
Supplement: TABLE S2 [file mbio.00676-22-st002.docx]

| **Bacterial strains** | **Description** | **Reference** |
| --- | --- | --- |
| ***E. coli*** |  |  |
| MC1061 | Laboratory cloning strain | (1) |
| ***S. pyogenes*** |  |  |
| HKU16 | Hong Kong *S. pyogenes emm*12 scarlet fever isolate | (2) |
| HKU16∆*gshT* | HKU16∆*gshT* isogenic mutant strain | This study |
| HKU16∆*gshT*++ | HKU16∆*gshT*::*gshT*-complemented strain | This study |
| **Plasmids** |  |  |
| pLZts | Temperature-sensitive shuttle plasmid, spectinomycin^R^ | (3) |
| pLZts-*gshT*_KO | pLZts+*gshT* knockout construct | This study |
| pLZts-*gshT*_complemented | pLZts+*gshT* complementation construct | This study |
| **Primers** |  |  |
| ***Mutagenesis*** |  |  |
| *gshT*_KO-S-F | ttggtcgtcagactgatgggccccggcaaatgtacgtacgg |  |
| *gshT*_KO-S-R | gaagtttcaatcctaaatattgttttttaatcatcctatc |  |
| *gshT*_KO-AS-F | atatttaggattgaaacttcctacagctaac |  |
| *gshT*_KO-AS-R | cataacctgaaggaagatctagaaattatagtgttggaagg |  |
| ***Quantitative real-time PCR*** |  |  |
| qRTPCR-*gyrA*-F | cgacttgtctgaacgccaaa |  |
| qRTPCR-*gyrA*-R | gtcagcaatcaaggccaaca |  |
| qRTPCR-*cysK*-F | cgatgattgaagctgctgaa |  |
| qRTPCR-*cysK*-R | agcacctacccatgcaagac |  |
| qRTPCR-*pncA*-F | atgatccttggcatcctgag |  |
| qRTPCR-*pncA*-R | agtccccgaaaaagctgaat |  |
| qRTPCR-*lacD.*1-F | tttgagtgcaggcgtatctg |  |
| qRTPCR-*lacD.*1-R | gaagccttcagtgcatagcc |  |
| qRTPCR-*manEIIC*-F | ttgatttgttttgggggaaa |  |
| qRTPCR-*manEIIC*-R | caggctcagctgcaataaca |  |
| qRTPCR-*arcA*-F | tatttcacacgggacccatt |  |
| qRTPCR-*arcA*-R | cttcaatgcgagtggtttca |  |
| qRTPCR-*lctO*-F | aagtagcgtctgccaaagga |  |
| qRTPCR-*lctO*-R | agctttgcaaccttgtgctt |  |
| qRTPCR-*slo*-F | caaagcaacggttgaggtca |  |
| qRTPCR-*slo*-R | gcaggaagcgtattaccacc |  |
| qRTPCR-*speB*-F | tgctgacggacgtaacttct |  |
| qRTPCR-*speB*-R | ccaccagtaccaagagctga |  |
| qRTPCR-*spyCEP*-F | caagacaacgacctcagcaa |  |
| qRTPCR-*spyCEP*-R | tttcggctaaagacgcaagt |  |
| qRTPCR-*sdaB*-F | acagggacacgtacccaaaa |  |
| qRTPCR-*sdaB*-R | cacgacagctcttggaatca |  |

**References**

1. Wertman KF, Wyman AR, Botstein D. Host/vector interactions which affect the viability of recombinant phage lambda clones. Gene. 1986;49(2):253-62.

2. Tse H, Bao JYJ, Davies MR, Maamary P, Tsoi H-W, Tong AHY, et al. Molecular characterization of the 2011 Hong Kong scarlet fever outbreak. J Infect Dis. 2012;206(3):341-51.

3. Barnett TC, Daw JN, Walker MJ, Brouwer S. Genetic manipulation of group A *Streptococcus*-gene deletion by allelic replacement. Methods Mol Biol. 2020;2136:59-69.
